# High-throughput multiplexed fluorescence-activated droplet sorting

**DOI:** 10.1038/s41378-018-0033-2

**Published:** 2018-10-22

**Authors:** Ouriel Caen, Simon Schütz, M. S. Suryateja Jammalamadaka, Jérémy Vrignon, Philippe Nizard, Tobias M. Schneider, Jean-Christophe Baret, Valérie Taly

**Affiliations:** 1 INSERM UMR-S1147, CNRS SNC5014, Paris Descartes University, Equipe labellisée Ligue Nationale contre le cancer, Paris, France; 2 Emergent Complexity in Physical Systems Laboratory (ECPS), Ecole Polytechnique Fédérale de Lausanne, 1015 Lausanne, Switzerland; 3 CNRS, University Bordeaux, CRPP, UMR 5031, 115 Avenue Schweitzer, 33600 Pessac, France

## Abstract

Fluorescence-activated droplet sorting (FADS) is one of the most important features provided by droplet-based microfluidics. However, to date, it does not allow to compete with the high-throughput multiplexed sorting capabilities offered by flow cytometery. Here, we demonstrate the use of a dielectrophoretic-based FADS, allowing to sort up to five different droplet populations simultaneously. Our system provides means to select droplets of different phenotypes in a single experimental run to separate initially heterogeneous populations. Our experimental results are rationalized with the help of a numerical model of the actuation of droplets in electric fields providing guidelines for the prediction of sorting designs for upscaled or downscaled microsystems.

## Bioanalysis: microfluidic-sorters to make better choices

Droplet-based microfluidics provides new technologies for cell screening by steering cell-containing microdroplets of interest into different channels at the kilohertz frequency. Until now, in conventional microfluidic sorting, droplets are sorted using electric pulses to move them into one of the two channels. Jean–Christophe Baret from the CNRS and University of Bordeaux in France, Valérie Taly from the CNRS and University of Paris Descartes and Tobias Schneider from EPFL in Switzerland have now boosted dielectrophoretic droplet manipulation to allow for multiple selections in a single device. The teams show how emulsified dye-loaded microdroplets are sorted in five different channels depending on fluorescence intensity: a computer algorithm controls the application of electric pulses to direct the droplets into one of the five channels depending on the fluorescence signals. Numerical models unravel the effect of the dielectrophoretic actuation on the droplet trajectories and are now used to predict the sorting conditions for droplet volumes compatible with single-cell encapsulation.

## Introduction

Fluorescence-based cell sorting is essential in numerous biological assays requiring high-throughput analysis and sorting of single cells. The gold standard technology for this purpose is fluorescence-activated cell sorting (FACS)^1^. However, a main drawback of FACS is that it cannot support real-time analysis of single cell or integration of complex assays involving single-cell manipulation, treatment, and final detection^2^. Moreover, it is not compatible with the analysis of small cell populations (< 10^5^ cells^3^). Compared with FACS, fluorescence-based cell sorting microsystems allow to reduce sample amounts to eliminate potentially biohazardous aerosols^4^ and to implement complex assays. A large variety of such microdevices has been developed in recent years, based on different physical mechanisms such as optical manipulation^5^, mechanical systems^6^, acoustophoresis^7^, and electrokinetics^8^.

Whereas both FACS and the latter systems are efficient to screen compounds remaining within the cell or on its surface, they are not suited for cytoplasmic or secreted proteins screening. Even though intracellular staining flow cytometry has been described, the use of protein transport inhibitors can interfere with the analysis^9^.

An alternative solution consists in the compartmentalization of cells in monodisperse emulsion droplets. A high viability of encapsulated cells in such droplets has been observed over several days^10^, and various active droplet sorting solutions such as acoustic, magnetic, pneumatic, thermal, and electric actuation have already been described^11^. However, the most widespread method is dielectrophoretic-based sorting.

The former approach has indeed been democratized for several reasons. First, dielectrophoresis is very efficient for the sorting of water-in-oil droplets, as it is mainly governed by the dielectric contrast between water and oil, independently of additives in these phases. Second, it allows the sorting of a large range of droplet volumes: from 20 fL^12^ to 10 nL^13^. Third, it allows to reach sorting rates as high as 30 kHz^14^, comparable with rates achieved by commercially available flow cytometers. Eventually, it is not restricted to fluorescence-based actuation, but can be peformed through absorbance^15^ or morphological^16,17^ measures. As such, the application scope of biological assays which can be performed through dielectrophoretic sorting is very broad. In particular, dielectrophoretic fluorescence-based sorting has already demonstrated a great interest for numerous biological applications. It has been used to screen enzymes expressed intracellularly^18,19^, on the surface of cells^20,21^ or secreted from cells^13,22,23^, as well as for the directed evolution of enzymes^20,24–26^. It has also been used to screen for extracellular metabolite production or consumption^27^ and to screen cells for monoclonal antibody production^28,29^. Eventually, it allowed to perform genetic sequence-specific sorting and to recover cell genomes for downstream nucleic acid analysis^30–32^.

However, in all former studies, sorting could be performed solely on two droplet populations, allowing to isolate a single phenotype. Indeed, despite both multi-parametric screening^33–37^ and multiplexed sorting^38–40^ have been demonstrated by FACS, mainly multi-parametric screening has been shown by droplet fluorescence-based microsystems^41–45^. So far only a single study showed the dielectrophoretic sorting of multiple (triple) fluorescent droplet populations^46^. However, this work consisted in serially integrating binary sorters and was limited to a throughput of 2–3 droplets per second. Here, we demonstrate the use of a single dielectrophoretic sorter, allowing to sort up to five different fluorescent droplet populations at rates of several hundreds of droplets per second.

## Materials and methods

### Microfluidic devices preparation

**Fig. 1:**
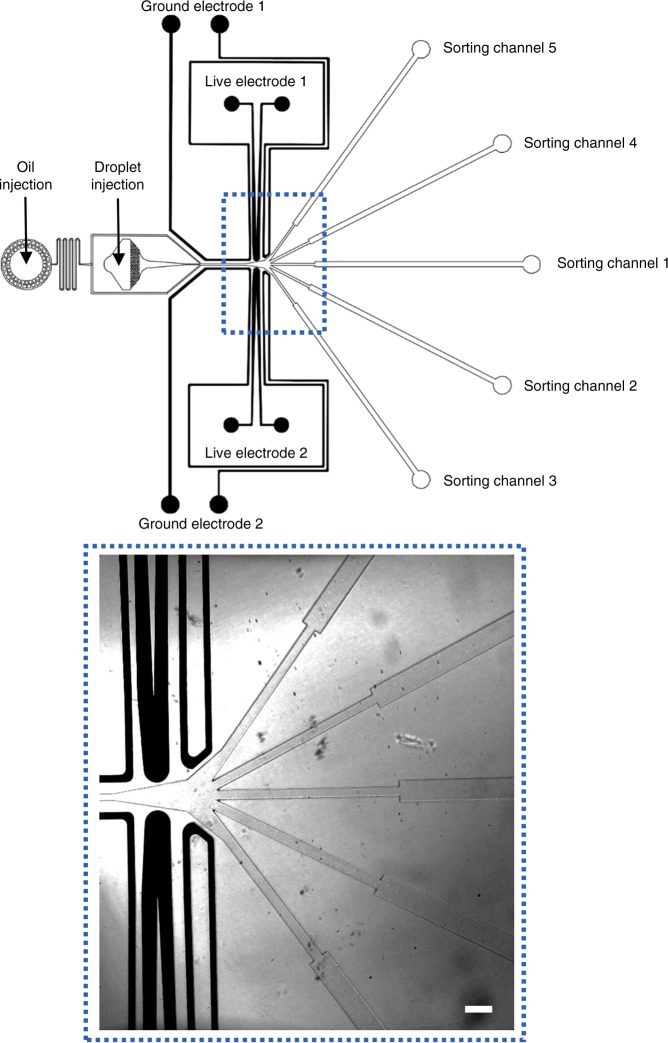
Design of the multiplexed droplet sorter. **Top**: design of the whole device. Droplets can be reinjected in the sorter and precisely spaced by oil for further sorting. Two symmetrical live and ground electrodes allow to apply an electric field above and below the central channel. Droplets can hence be deviated into one of the four channels in periphery or remain in the central channel in the absence of electric actuation. **Bottom**: inset of the sorting zone. Scale bar: 200 μm

The following protocol is similar to the one we previously described in ref ^47^. The microfluidic devices were designed with conventional computer-aided design (CAD) tool (AutoCAD, AutoDesk) and printed on an optical grade transparent film (Selba). The design of the sorting device can be found in Fig. 1 and the design of the droplet production device can be found in Fig. S1. Molds of SU-8 negative photoresist (MicroChem) were fabricated on a silicon wafer (Si-Mat) by UV exposure (MJB4 contact mask aligner; SUSS MicroTec) through the photolithography mask (Selba) and developed (SU-8 developer; Micro-Chem). SU8-2025 was used to prepare a 35-μm deep mold for the droplet production device, and SU8-2035 was used to prepare a 50-μm deep mold for the sorting device. Curing agent was added to the poly (dimethylsiloxane) (PDMS) base (Sylgard 184 silicone elastomer kit; DowCorning) to a final concentration of 10% (w/w), mixed, and poured over the mold. Following degassing for 20 min and cross-linking at 75 °C for 1 h, the PDMS was peeled off the mold and the input and output ports were punched with a 0.75-mm diameter biopsy punch (WPI). The holes for the sample loading wells of the droplet production device were punched by using a biopsy puncher with a 5 mm diameter (Harris Uni-Core). Particles of PDMS were cleared from the ports using Scotch tape, rinsing with isopropanol, and drying with pressurized nitrogen. The structured side of the PDMS slab was bonded to a 75 × 50 × 1.2-mm glass microscope slide (Corning) by exposing both parts to an oxygen plasma (PICO, Diener) and pressing them together. Finally, an additional hydrophobic surface coating was applied to the microfluidic channel walls by injecting the completed device with Aquapel glass treatment (PPG Industries) and then purging the liquid with nitrogen gas. For the sorting chips, the PDMS device was plasma bonded to the non-conductive side of a 75 × 25 × 1.1-mm indium tin oxide glass (ITO, Delta Technologies). The conductive side of the ITO glass was used as a counter electrode. Electrodes were incorporated into the system by filing the micro channels with a low-melting point solder (Indalloy 19, Indium corporation).

### Fluid actuation

Droplets were produced by flow focusing the aqueous phase with a fluorinated oil phase (HFE7500, 3 M) containing 2% (w/w) EA surfactant (RainDance Technologies), a biocompatible PEG–PFPE amphiphilic blockcopolymer^48^. A red fluorescent dye, Sulforhodamine B (Sigma-Aldrich), was added to the aqueous phase (PBS water) to fluorescently label the droplets. Aqueous and oil phases were injected into the microfluidic chips using an MFCS pressure controler (Fluigent). For droplet selection at a 80 Hz rate, droplet flow rate was ≈0.23 μL/min and oil flow rate was 8.1 μL/min. For droplet selection at a 450 Hz rate, droplet flow rate was ≈1.3 μL/min and oil flow rate was 50 μL/min. Oil flow rates were monitored using a syringe pump controler (neMESYS, Cetoni). The droplet library preparation for the fluorescence-based multiplexed droplet sorting was performed as follows. First, five different aqueous phases were prepared at five concentrations of Sulforhodamine B (9.37, 18.75, 37.5, 75, and 150 μM) diluted in PBS water. These five solutions were independently loaded into a dedicated open well within the microfluidic chip. The device was embedded in a pressure chamber, previously described in ref ^31^, such that each well was pressurized at 500 mbar. These aqueous phases were independently flow focused by oil, which was pressurized at 1 bar. The generated droplet library was then reinjected into the sorting device. Droplets were pressurized at 100 mbar and spaced by an oil phase pressurized at 373 mbar. The central collection channel was connected to a polytetrafluoroethylene (PTFE) tubing (Fisher Scientific) with an internal diameter (ID) of 0.56 mm. Other collection channels were connected to polyetheretherketone (PEEK) tubings (CIL Upchurch) with ID of 0.25 mm.

### Principle for multiplexed sorting

Traditionally, droplet sorting is performed on two distinct droplet populations. In the absence of an external actuation, the trajectory of the droplets in the channels is driven by hydrodynamics. Typically, there are two outlet channels, with one arm wider than the other. Such a geometry allows a contrast of hydrodynamic resistance between the two arms. Droplets thus follow the main flow toward the less-resistant arm in the absence of an external field. When an external electric field is applied, the droplets are forced across the streamlines to flow toward the other arm. We consider here the case of a dielectric droplet of dielectric constant *ε*_*d*_ immersed in a perfect dielectric material of dielectric constant *ε*, where *ε*_*d*_ > *ε*. For simplicity, we use the same constant to describe the continuous phase fluid and the polymer in which the channels are molded. The field oscillates at high frequency (30 kHz) to avoid the accumulation of free charges. Typically, the charge relaxation time of a water droplet is 0.125 ms^49^, therefore smaller than 30 kHz. As no free charges are present in the system, the stress on the droplet interface is given by the jump in Maxwell stress^50^,

1 $$\vec f = \frac{{\varepsilon _0\varepsilon }}{2}\left[ {\left( {1 - \frac{\varepsilon }{{\varepsilon _d}}} \right)E_n^2 - \left( {1 - \frac{{\varepsilon _d}}{\varepsilon }} \right)E_t^2} \right]\vec n,$$

where $\vec n$ is the interface normal pointing outside the droplet and *E*_*n*_ (*E*_*t*_) is the normal (tangential) field component in the continuous phase at the interface. Integrating this stress over the droplet surface gives the net dielectrophoretic force, which is independent of the sign of the field and will be attractive toward regions of high field strength. A common approximation of this force, for the case of a spherical droplet in a weakly varying field, is given by the expression:

2 $$\vec F_{dep} = 2\pi \varepsilon _0\varepsilon K\left( {\varepsilon ,\varepsilon _d} \right)R^3\nabla \left| {\vec E} \right|^2,$$

where *K*(*ε*, *ε*_*d*_) = (*ε*_*d*_ − *ε*)/(*ε*_*d*_ + 2*ε*) is the Clausius–Mossotti factor, *R* the droplet radius, and $\vec E$ the electric field. In the general case, the net force depends on both the droplet shape and the electric field in the presence of the droplet. With no general analytical solution for this problem, numerical simulations provide the proper value and distribution of the force. The dielectrophoretic force scales with the square of the potential difference between the live electrode and the ground electrode. Traditionally, a constant value of potential difference is chosen such that the DEP force is high enough to deviate the droplet in the relevant arm. Here we propose to use a symmetric multidirectional sorter, and vary the potential difference or actuation time between the electrodes such that various amplitudes of DEP forces can be applied. The droplet deflection can be changed and thus allow to sort droplets toward several different channels.

### Sorting design

The implementation of the sorting design is presented in Fig. 1. In brief, an emulsion is reinjected in the central channel and spaced by oil. On each side of the channels, a live electrode surrounded by two ground electrodes used for electrical shielding is disposed in a geometrical configuration, allowing to maximize the dielectrophoretic force applied at the sorting junction. The electrodes at the sorting junctions are independently controlled by 30 kHz sinusoidal voltages amplified to a high voltage (0–1200 Vpp) using a set of two amplifiers (Trek, 623B). Such a frequency is more than an order of magnitude faster than the time scale in our system, such that any forces due to electrostatics would average to zero over multiple oscillations. At the junction, five separate outlets are designed to collect the five fractions of the droplet, depending on their fluorescence intensities. No voltage was applied to sort the droplets in the middle channel. Electrode activation was synchronized with droplet detection through the Labview FPGA interface at 200 kHz. An oil injection inlet integrated in the sorter design (see Fig. 1) allowed to homogeneously space the droplets while they were reinjected in the sorting device. For droplet selection at a 80 Hz rate, voltage amplitude applied to the exterior channels (numbers three and five) and interior channels (numbers two and four) was, respectively, 1200 and 900 Vpp, with an electrode activation time of 1.7 ms. For droplet selection at a 450 Hz rate, voltage amplitude applied to the exterior channels (three, five) and interior channels (two, four) was 800 Vpp, with electrode activation times of 3.3 and 1.17 ms, respectively. For multiplexed fluorescence-based sorting, voltage amplitude applied to the exterior channels (three, five) and interior channels (two, four) was, respectively, 1200 and 900 Vpp, with an electrode activation time of 1 ms.

### Optical detection

The microfluidic chip was interfaced on a previously described setup for fluorescence measurements^47^. In brief, the droplets continuously flow through an excitation laser (Cobolt 06-DPL 532 nm; Cobolt), and their emitted fluorescence was detected by a photomultiplier tube (PMT) (Hamamatsu H5784-20) in the range of 560–590 nm. A detailed description of the used optical setup has been previously reported^47^. A voltage proportional to the intensity of the emitted light and therefore to the dye concentration is then measured, recorded, and statistically analyzed. The sorting decision was programmed in a labview routine to select up to five different ranges of intensities.

### Data acquisition and analysis

All videos were recorded with a high-speed digital camera (Phantom Miro M310, Vision Research). A Hough circled detection algorithm^51^ was used to roughly localize droplets centers and sizes. This rough detection was then refined using Virtual Image Correlation^52^ based on a gray-level distribution along one cross-section of the droplet shape. This refinement allowed for a sub-pixel estimation of the 2D droplet section imaged by the high-speed camera. Such analysis allowed to monitor droplet trajectories and volumes. The data acquisition and control procedure of optical and triggering signals is similar to the one we previously described in ref ^47^. The PMT signal was recorded and converted to 8 bits through the Analog to Digital Converter of a LabVIEW FPGA module. The sampling frequency was adjusted to 200 kHz. At each acquisition step, 8-bit PMT values were joined to form a 64-bit word queued in a LabVIEW direct memory access (DMA) first in, first out (FIFO). DMA FIFO enables high bandwidth transfer of data from the field-programmable gate array (FPGA) to the host computer. Three 8-bit signals were joined in these 64-bit words, such that PMT acquisition could be transferred together with the signals of high-voltage signal triggers. On the host computer, FIFO elements were dequeued in the main LabVIEW vi. Each 64-bit word was decoded to the three original 8-bit values. Data were then directly streamed to the disk in a Waveform Audio FileFormat (WAV) 8-bit PCM. An open-source digital audio editor (Audacity) was used to convert the signal into 16 bits format, and a home-made MATLAB script was further used to analyze the signals.

### Numerical simulations

For the numerics, we used a 3D boundary element method (BEM)^53^ on quadrilateral linear elements. Given the potential *φ* on the electrode surfaces and coupling the solutions in the droplet and the continuous medium by matching the potentials and normal components of the displacement field $\vec D_i = \varepsilon _i\vec E_i$ on the droplet interface, the electrostatic Maxwell equation

3 $${\mathrm{\Delta }}\varphi = 0$$

was solved for the flux $\vec n \cdot \nabla \varphi$ on the electrode surfaces, and flux and potential on the droplet interface. From this, the field on the droplet surface was constructed. The resulting Maxwell stress (1) was added to the stress due to surface tension,

4 $$\vec f_\gamma = - 2\gamma \kappa \vec n$$

where *γ* is the surface tension and *κ* the mean curvature of the surface, to couple the inner and outer solution for a BEM-solver of the Stokes equations,

5 $$\nabla \cdot \vec u = 0$$

6 $$\nabla \cdot \hat \sigma = \vec 0$$

where $\vec u$ is the flow velocity and $\hat \sigma$ the Newtonian stress tensor. Coupling at the interface took into account the different viscosities of the fluids. After solving for the interface velocity, the interface was advected in an explicit Euler time step. This process was repeated until droplets had reached the outlet of the sorter. Mesh regularity was enforced with an active redistribution of interface vertices and a quadtree-based mesh refinement. Mesh handling and surface integration were implemented based on the library deal.II^54^, solving the linear system used Jacobi-preconditioned GMRES.

The numerical code was successfully validated in a series of tests, details can be found in ref ^55^. The simulation reproduced Stokes’ law for the drag force on a spherical particle with a relative error of order 10^−7^ and the dielectric drag force on a spherical droplet due to a nearby point charge with a relative error of order 10^−2^. Both the dynamic deformation of droplets due to viscous shear^56^ and electric fields^50^ was reproduced with relative errors of order 10^−2^. No fitting parameters were used.

## Results and discussion

### Droplet selection

As a first step, we measured the efficiency of our design to selectively drive the droplets in the different channels. In the absence of electric field, the droplets flow to the central channel (Fig. 1, sorting channel 1) since the flows are laminar and the channel is designed with a reflection symmetry axis. Two decoupled live electrodes from each side of the central channel allow electric actuation to drive the droplets above and below the central channel (Fig. 1).

The lower electrode is activated to sort the droplets to the lower interior channel and the lower exterior channel (see Fig. 1, sorting channel 2 and sorting channel 3, respectively). Similarly, the upper electrode is activated to drive the droplets to the upper interior channel and the upper exterior channel (Fig. 1, sorting channel 4 and sorting channel 5, respectively). The width of the sorting channels was designed larger than droplets diameter (75 μm vs. 45 μm, respectively) to prevent friction between the droplets and channel walls. For the same reason, the depth of the sorting device was chosen to be 50 μm. As the distance between central and exterior channels is larger than the one between central and interior channels, it is expected that higher voltages or higher actuation times are necessary to carry droplets in the exterior channels. Since high voltages could result in droplet electrosplitting, it is of interest to minimize the voltage needed to deflect droplets in exterior channels. This can be either achieved by increasing the electrode activation time and lowering the hydrodynamic resistance of these channels. For the latter purpose, we broadened the entrances of exterior channels, and their width was designed 10 μm larger than other channels. These modifications did not affect the behavior of droplets in the absence of an external field given the symmetry of the sorter.

**Fig. 2:**
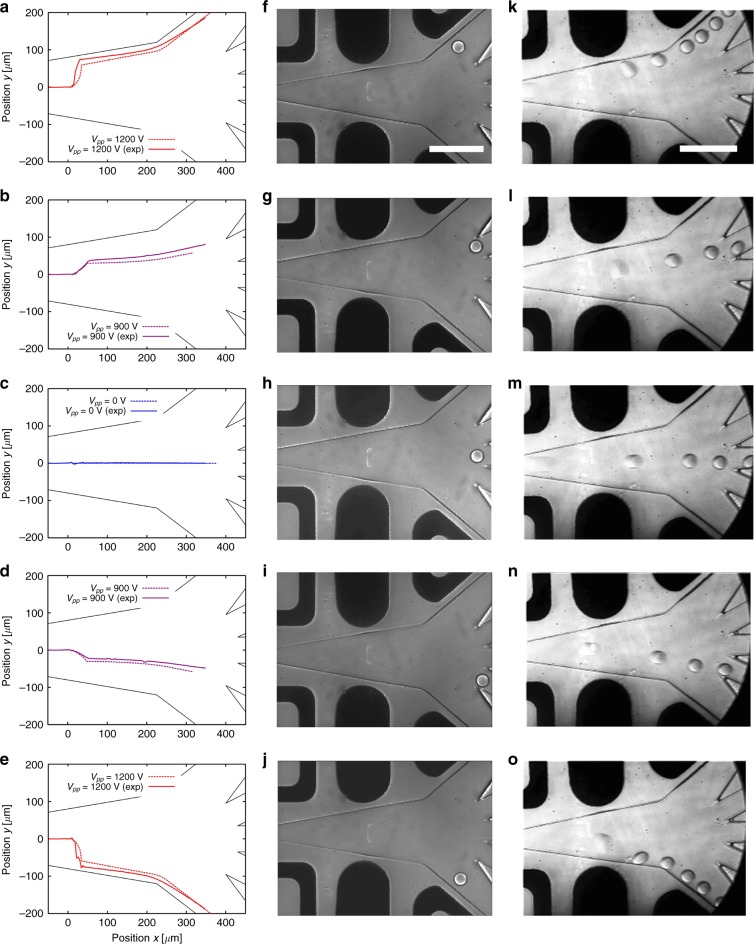
Droplet deflections into the sorting channels. **a**–**e** Droplet trajectories tracked using a home-made image analysis software (*n* = 600 droplets for each trajectory). Simulated trajectories are shown in dashed lines. Droplets deflection rate was 80 Hz with a droplet flow rate of ≈0.23 *μ*L/min and an oil flow rate of 8.1 μL/min. Voltage amplitude applied to the exterior channels and interior channels was, respectively, 1200 and 900 Vpp, with an electrode activation time of 1.7 ms. No voltage was applied to sort droplets in the middle channel. **f**–**j** Corresponding microscopy images of droplet deviation at 80 Hz (see Movies S1–5, ESI). Scale bar: 200 μm. **k**–**o** Microscopy images of droplet deviation at 450 Hz (see Movies S6–10, ESI). Droplet flow rate was ≈1.3 μL/min and oil flow rate was 50 μL/min. Voltage amplitude applied to the exterior channels and interior channels was 800 Vpp, with electrode activation times of 3.3 and 1.17 ms, respectively. No voltage was applied to sort droplets in the middle channel. Scale bar: 200 μm

A 30 kHz sinusoidal voltage was generated and amplified to be applied to the electrodes connected to the chip. We calibrated the applied voltages and electrode activation times to lead the droplets into the targeted sorting channels (see Fig. 2). As expected, higher voltages or actuation times were required to direct the droplets to the exterior channels than those necessary to allow deflection into interior channels. We were able to reliably deflect the droplets in the relevant sorting channels for throughputs ranging from 80 to 450 Hz (see Fig. 2 and Movies S1–10 in the ESI).

As a next step, we compare the experimental trajectories of the droplets in the external field to numerical simulations in order to confirm the actuation mechanism. A high consistency was shown between experimental and simulated droplet trajectories (see Fig. 2a–e). The slight mismatches observed between experimental data and simulations can have several sources. From an experimental perspective, they can originate from image analysis noise and variations in the channel thickness. As in regard to the numerics, limited spatial and temporal resolution of the simulation scheme could be responsible for inaccuracies. We conclude from these results that our numerical scheme can indeed be used as an efficient predictor of the droplet behavior in an external electric fields, providing the basis to the rational design of sorting junctions.

### Multiplexed droplet sorting

**Fig. 3:**
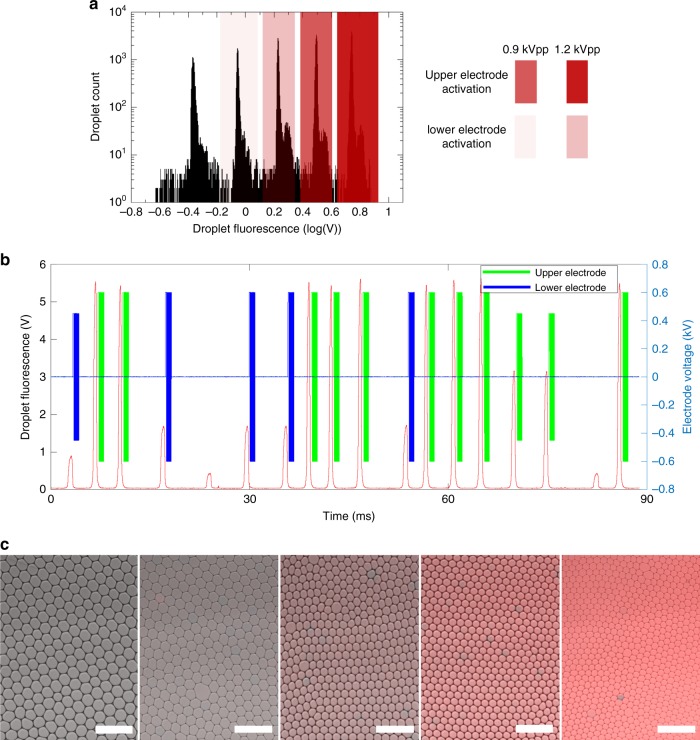
Automated fluorescence-based droplet sorting. **a** Fluorescence histogram of the droplet library, *n* = 86,430 droplets. Sorting gates selecting the different fluorescent droplet populations for electric actuation are highlighted as red rectangles. **b** Time series showing sorting triggers for different members of the droplet library. Sorting actuation efficiency was higher than 98.4%, *n* = 62,746 droplets. **c** Fluorescence microscopy images of sorted droplets. Left to right: droplet populations 1–5 are respectively represented with corresponding fluorescence intensities (mean ± s.d. [a.u]): 4.1 ± 0.01, 10.6 ± 0.01, 31.2 ± 1.1, 46.5 ± 0.7, and 112.3 ± 1.7. Scale bar: 200 μm (droplets volume is 48 pL in all images, variability is due to the imaging conditions)

In a final step, we automated the sorting using a home-made LabVIEW FPGA software. A library of five different droplet populations was generated and encoded by different concentrations of a red fluorescent dye (Sulforhodamine B). Droplet monodispersity is a fundamental parameter to guarantee highly quantitative assays. As such, in order to ensure a high monodispersity of the droplet library, we used a previously developed multiplexed emulsifier to generate droplets of 45 μm diameter^31^ (see Fig. S1). Such accuracy in droplet volume is essential for the robustness of sorting for several reasons. First, it ensures a high spectral resolution of the droplet library allowing to properly gate each droplet population (see Fig. 3a). Second, it guarantees that droplets are homogeneously spaced during their reinjection into the sorter such that each droplet can be triggered within the same time delay. Finally, it allows for each droplet to consistently be deflected with the same angle at a given applied voltage.

Once generated the droplet library was reinjected for sorting at a rate of 200 Hz (see Movie S11, ESI). The oil phase reservoir was pressurized at 373 mbar and the droplet phase reservoir at 100 mbar. A histogram of droplets fluorescence levels could thus be plotted in real time within the software interface. The software allowed for each member of the droplet library to automatically be sorted in a dedicated sorting channel based on its fluorescence concentration. Sorting triggers were then automatically applied for each droplet depending on its fluorescence concentration (see Fig. 3b). Each individual fraction is recovered and plated for imaging: the droplets clearly exhibit distinct fluorescence levels for each sorted fraction (see Fig. 3c). The success rate of sorting actuation was higher than 98.4%. This rate was determined as follows. For each gated droplet fluorescence population, we statistically analyzed the specifications of the corresponding electrical trigger (electrode actuation, electrode position, and voltage amplitude), which was generated during sorting for each droplet. For more than 98.4% of 62,746 analyzed events, the generated trigger was consistent with the expected one. Actuation errors were due to instabilities during droplets reinjection which resulted in irregular spacing between the droplets at the sorting junction. Such irregular spacing is due to coalesced droplets. Droplet coalescence was minimized by the use of surfactants in the oil phase, filtering of reagants, and gentle experimental handling.

### Scaling relationships

**Fig. 4:**
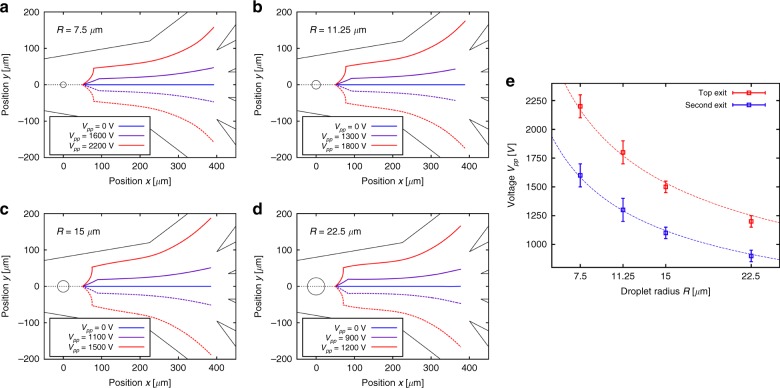
Scalability of multiplexed sorting for different droplet sizes. **a**–**d** Simulation results for sorting the droplets into the different channel exits, for droplets of different radius *R*. The total flow rate was 8.1 μL/min and the electrode activation time was 1.7 ms. **e** Simulation results for the peak-to-peak voltage *V*_*pp*_ required to steer the droplet into each of the channel exits, for different droplet radii *R*. The dashed lines give the relation $V_{pp} \propto 1{\mathrm{/}}\sqrt R$

The combination of our experiments and a reliable numerical method to solve the flow of droplets in external fields is finally used to discuss the scaling properties of our sorting junction. We also demonstrated numerically that our sorting design could be used to sort a wide range of droplet volumes (spreading over an order of magnitude) by properly calibrating applied voltages (see Fig. 4). For the range of droplet radii studied, the voltage Vpp required to steer droplets of radius *R* into the exits scales with $V_{pp} \propto 1{\mathrm{/}}\sqrt R$. Due to the influence of the top and bottom walls, the drag force is approximately proportional to^57^

7 $$F_{Drag} \propto R^2U_{Drift}$$

for *R* in the range we consider. Since the dielectrophoretic force (2) scales with

8 $$F_{DEP} \propto R^3V_{pp}^2,$$

the drift velocity *U*_*Drift*_ has the scaling

9 $$U_{Drift} \propto RV_{pp}^2$$

and is therefore constant if $V_{pp} \propto 1{\mathrm{/}}\sqrt R$.

## Conclusions

In summary, we have designed a new type of microfluidic sorter for multiplex sorting of droplet libraries. Our strategy is based on the most widely adopted technology for fluorescence-based droplet sorting, which is dielectrophoretic actuation. We show that up to five fractions can be isolated in one single run at a high-throughput, up to 200 droplets per second. We therefore overcome here the limitation of traditionally bidirectional sorter which restrict their use to the separation of two droplet populations. Such a sorting system could be valuable to sort cells with different phenotypes in a single round, through either fluorescence-, absorbance-, or morphological-based actuation. The comparison of our experiments and numerical simulations validate the physical ground of the droplet actuation model. We can now predict with numerical simulations the properties of the sorter for different droplet sizes. We believe that this numerical approach will be useful in the long run to rationally design devices and go beyond the trial-and-error methods used until now.

## Electronic supplementary material

Supporting information

Movies S1 to S11
