# Supplementary material for: High-throughput multiplexed fluorescence-activated droplet sorting
Source: Microsyst Nanoeng. 2018 Oct 22;4:33. doi: 10.1038/s41378-018-0033-2 (PMC6220162; doi:10.1038/s41378-018-0033-2)
Supplement: Supplementary file 1 — Supporting information [file 41378_2018_33_MOESM1_ESM.pdf]

High throughput multiplexed fluorescence activated droplet sorting

Ouriel Caen,[1] Simon Schütz,[2] Mani S.S. Jammalamadaka,[1] Jérémy Vrignon,[3] Philippe Nizard,[1] Tobias M. Schneider,[2,*] Jean-Christophe Baret,[3,] and Valérie Taly[1,***]**

[1] INSERM UMR-S1147, CNRS SNC5014, Paris Descartes University, Equipe labellisée Ligue Nationale contre le cancer, Paris, France
[2] Emergent Complexity in Physical Systems Laboratory (ECPS), Ecole Polytechnique Federale de Lausanne, 1015 Lausanne, Switzerland
[3] CNRS, Univ. Bordeaux, CRPP, UMR 5031, 115 Avenue Schweitzer, 33600 Pessac, France
*tobias.schneider@epfl.ch
**jean-christophe.baret@u-bordeaux.fr
***valerie.taly@parisdescartes.fr

Supplementary Information

Supplementary Figure S1

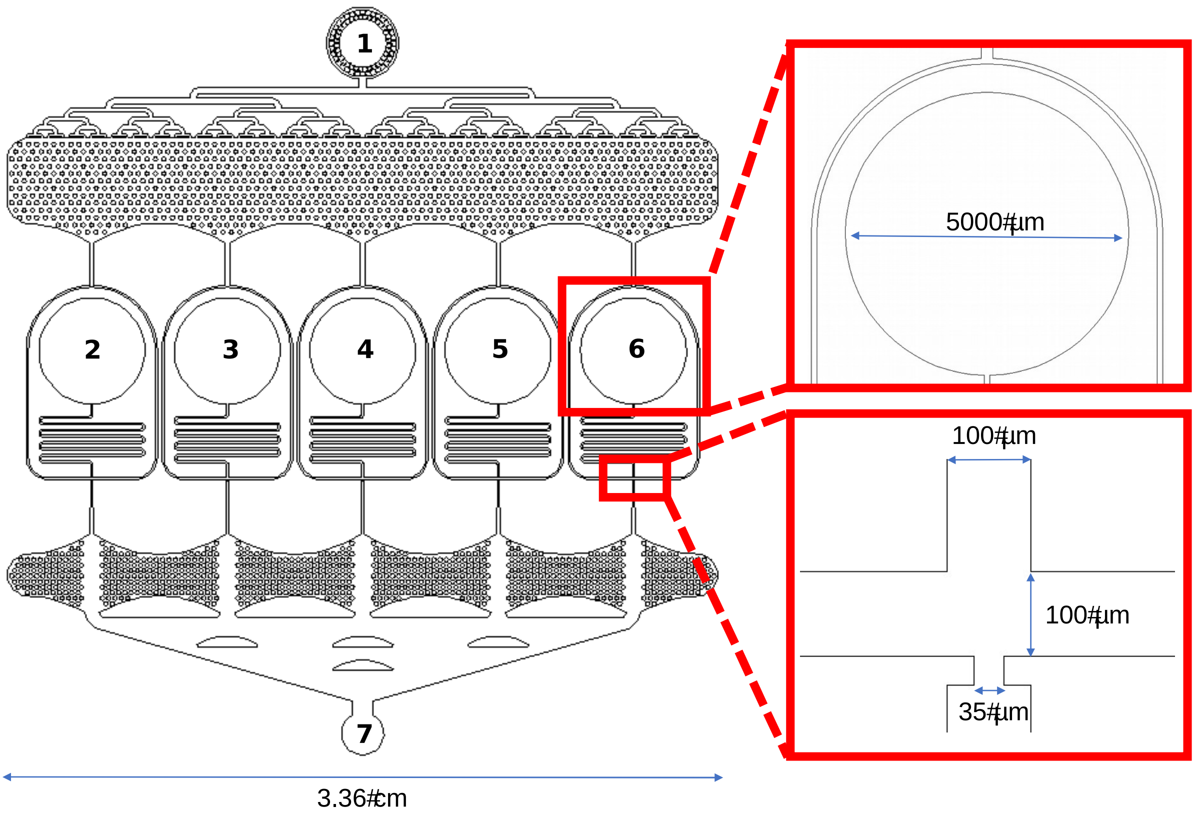

Figure S1. Design of the microfluidic chip used to parallely emulsify five fluorescently encoded droplet populations. 1: Oil inlet. 2-6: five open wells to deposit independent aqueous phases. 7: Droplet collection outlet.

Supplementary Table S1

Droplet population/targeted sorting channel n°	1	2	3	4	5
Droplet fluorescence concentration (µM)	9.37	18.75	37.5	75	150
Voltage actuation through lower electrode (Vpp)	0	900	1200	0	0
Voltage actuation through upper electrode (Vpp)	0	0	0	900	1200

Table S1. Sum up of the protocol to sort five different fluorescent droplet populations according to a specific actuation voltage.

Supplementary Video 1

Movie S1. Droplet deviation into the upper exterior channel under electric actuation of the upper electrode (1.2 kVpp, 1.7 ms). Droplet selection rate was 80 Hz.

Supplementary Video 2

Movie S2. Droplet deviation into the upper interior channel under electric actuation of the upper electrode (0.9 kVpp, 1.7 ms). Droplet selection rate was 80 Hz.

Supplementary Video 3

Movie S3. Droplet deviation into the central channel in the absence of electric actuation. Droplet selection rate was 80 Hz.

Supplementary Video 4

Movie S4. Droplet deviation into the lower interior channel under electric actuation of the lower electrode (0.9 kVpp, 1.7 ms). Droplet selection rate was 80 Hz.

Supplementary Video 5

Movie S5. Droplet deviation into the lower exterior channel under electric actuation of the lower electrode (1.2 kVpp, 1.7 ms). Droplet selection rate was 80 Hz.

Supplementary Video 6

Movie S6. Droplet deviation into the upper exterior channel under electric actuation of the upper electrode (0.8 kVpp, 3.3 ms). Droplet selection rate was 450 Hz.

Supplementary Video 7

Movie S7. Droplet deviation into the upper interior channel under electric actuation of the upper electrode (0.8 kVpp, 1.17 ms). Droplet selection rate was 450 Hz.

Supplementary Video 8

Movie S8. Droplet deviation into the central channel in the absence of electric actuation. Droplet selection rate was 450 Hz.

Supplementary Video 9

Movie S9. Droplet deviation into the lower interior channel under electric actuation of the lower electrode (0.8 kVpp, 1.17 ms). Droplet selection rate was 450 Hz.

Supplementary Video 10

Movie S10. Droplet deviation into the lower exterior channel under electric actuation of the lower electrode (0.8 kVpp, 3.3 ms). Droplet selection rate was 450 Hz.

Supplementary Video 11

Movie S11. Fluorescence-based droplet sorting of five fluorescently encoded droplet populations (see conditions in Table S1). Droplet sorting rate was 200 Hz.
